# Innovation During a Pandemic: Developing a Guideline for Infection Prevention and Control to Support Education Through Virtual Reality

**DOI:** 10.3389/fdgth.2021.628452

**Published:** 2021-07-09

**Authors:** Nathan Moore, Kathy Dempsey, Peter Hockey, Susan Jain, Philip Poronnik, Ramon Z. Shaban, Naseem Ahmadpour

**Affiliations:** ^1^Research and Education Network, Western Sydney Local Health District, Sydney, NSW, Australia; ^2^Faculty of Medicine and Health, The University of Sydney, Sydney, NSW, Australia; ^3^Clinical Excellence Commission, Sydney, NSW, Australia; ^4^Faculty of Medicine and Health, School of Medical Sciences, The University of Sydney, Sydney, NSW, Australia; ^5^Faculty of Medicine and Health, Susan Wakil School of Nursing and Midwifery, University of Sydney, Camperdown, NSW, Australia; ^6^Marie Bashir Institute for Infectious Diseases and Biosecurity, University of Sydney, Camperdown, NSW, Australia; ^7^Division of Infectious Diseases and Sexual Health, Westmead Hospital and Western Sydney Local Health District, Westmead, NSW, Australia; ^8^New South Wales Biocontainment Centre, Western Sydney Local Health District and New South Wales Ministry of Health, Sydney, NSW, Australia; ^9^Design Lab, School of Architecture, Design, and Planning, The University of Sydney, Sydney, NSW, Australia

**Keywords:** virtual reality, head mounted display, clinical education, infection control, cleaning, disinfection, guideline

## Introduction

The COVID-19 pandemic has had significant impacts on the delivery of clinical education worldwide. While the effects on, and consequences for, the delivery of education has been unparalleled, the events have given rise to innovation with the adoption of new, and emerging technologies. Virtual Reality (VR) is one such platform which is being adopted more readily due to its ability to deliver flexible and immersive education.

The reduction in hardware costs over the past decade motivates the increasing attraction to VR technology for clinical education in both the undergraduate and postgraduate settings. The emergence of untethered standalone VR Head Mounted Display (HMD) systems, such as the Oculus Quest™ and Oculus Go™, has allowed users to borrow relatively inexpensive equipment to use at the time and place of their choosing.

Like most healthcare institutions worldwide, the emergence of COVID-19 resulted in the halting of much research and innovation as attention focused on the immediacy of providing clinical care to large numbers of patients. In the authors' primary Local Health District (LHD) this included the cessation of the nascent VR education programs as local Infection Prevention and Control (IPAC) teams identified an infection risk in the absence of safe cleaning and disinfection policies and procedures for HMDs. This is particularly important as the equipment is shared among students and clinicians in training in the clinical setting.

The rapid pace of adoption of these technologies in response to COVID-19 has meant that policies and procedures have not been developed to address identified or unperceived risks, particularly in infection prevention and control. There is a lack of operational guidance for cleaning and disinfection of VR HMDs to allow for safe transfer between clinicians. This paper documents the process of developing an operational guide for cleaning and disinfection of HMDs in New South Wales (NSW), Australia (1). We first describe the emergence of VR as a supporting modality for clinical education, the principles of infection prevention and control and describe how these principles underpinned the development of a guideline for VR cleaning and disinfection to ensure the safety of users. We then identify future directions for research and innovation.

## VR in Clinical Education

The combination of interaction, immersion and imagination in VR has made it an attractive platform for users in clinical education (2). Contemporary educational solutions that allow for the application of skills such as simulation-based learning, are established and effective ways to develop technical and non-technical skills (3). Despite the effectiveness of simulation as an educational modality, there are some limitations. Simulations require large resource commitments both in equipment and faculty resources, require fixed locations of either simulation labs or clinical settings as well as fixed times linked to instructor availability (4).

VR based training can possibly overcome these challenges in being potentially self-led in a time and place of the users choosing. An additional contemporary benefit to the portability of VR can be identified with the constraints placed on healthcare and academic institutions since the emergence of COVID-19. The need for education to be flexible, adaptable, and remotely accessible has become an essential component for effective delivery. The complex makes and model of VR equipment along with the VR headset positioning during use, pose unique challenges to effective cleaning and disinfection in between use. The cost of material and consumables coupled with complexity of healthcare environments and need for meticulous cleaning requirements with any shared items lead to the exploration of opportunities to improve the feasibility of these devices in healthcare settings.

As an example of the rapid innovation that recent events have been a catalyst for, the authors have developed several innovative VR based applications to supplement face-to-face education in the health service. VR based applications supporting Advanced Life Support (ALS) leadership called ALS-SimVR (5, 6), Code Black identification and response training and a tool for reflective practice in a variety of clinical and non-clinical settings are all in varying stages of development within the LHD.

With the procurement of VR hardware by hospitals and universities to deploy what might be described as “*education through VR*,” it is likely that further investment in application development will occur to take advantage of this existing infrastructure. Consequently, there will be increased sharing of these HMDs between users which may pose a potential risk from an infection prevention and control perspective. Our work aims to mitigate this risk through a feasible, scalable, cost-effective, and standardized procedure, which is grounded in the infection prevention and control principles, discussed next.

## Infection Prevention and Control Principles

Infectious diseases are transmitted from person to person by direct or indirect contact with a person or via contaminated objects (7). Recent evidence demonstrates Fomite contamination of pathogenic microorganisms as an important factor in the spread of COVID-19 and other coronaviruses such as porcine epidemic diarrhea virus, Middle East Respiratory Syndrome coronavirus and human coronavirus 229E and OC43 (8). Laboratory evidence suggests that the SARS-CoV-2 can survive on dry surfaces for days to weeks, particularly on non-porous surfaces (9, 10). These findings emphasize the need for adequate cleaning of shared equipment and surfaces and hand hygiene to prevent the spread of infection. COVID-19 has necessitated the need to explore innovative methods to conduct large scale and rapid education and training to replace, supplement or complement existing face-to-face opportunities. Maintaining the principles of infection prevention and control is key to ensuring VR remains an effective platform to provide safe and efficient education to health workers during the COVID-19 pandemic and beyond. However, the complex makes, and models of VR devices highlighted the challenges around effective cleaning and disinfection in between use to minimize the risk of transmitting pathogenic microorganisms between users.

There are a number of VR products available in the market with varying levels of instruction on cleaning and disinfection (11, 12). The different components of the HMD come into contact with the face and hair of the user; and the VR's controllers come into contact with users' hands. The proximity of VR equipment to a user's mucus membranes such as nose, mouth or eyes potentially increase the risk of cross transmission if these items are not cleaned and disinfected before use. These reusable items may be shared between different users in their home or in the healthcare setting, making the cleaning, and disinfection process of utmost importance.

## Developing a Guideline for VR Infection Control

We conducted a search using the MEDLINE and Embase database on the 10th of October 2020 restricted to publications from 1996-current using the keywords infection control, sterilization, disinfection, equipment contamination/or decontamination, disinfectants, virtual reality, cross infection and computers. Following this search and extensive communication with other experts in the field no specific published guidelines were identified to guide the cleaning and disinfection of HMDs.

Author NM approached authors KD & SJ from the NSW Health Clinical Excellence Commission for state-wide guidance and support in developing a set of guidelines to allow for the effective cleaning and disinfection of HMDs. In collaboration with the Clinical Excellence Commission, we developed a Cleaning and Disinfection of virtual reality equipment Guideline to enable safe and appropriate use of the VR equipment between users (13). The guideline would draw on existing infection prevention and control principles and IPAC team knowledge to deploy VR HMDs for clinical education where it is used by healthcare providers.

The complexity of VR equipment, compatibility with cleaning and disinfection products, and accessing difficult to clean parts are some of the considerations given when developing the guidance around appropriate management of these devices in health. To ensure appropriate hygiene is maintained in between uses, there are a number of practices employed to make the VR equipment safe.

**Figure 1:**
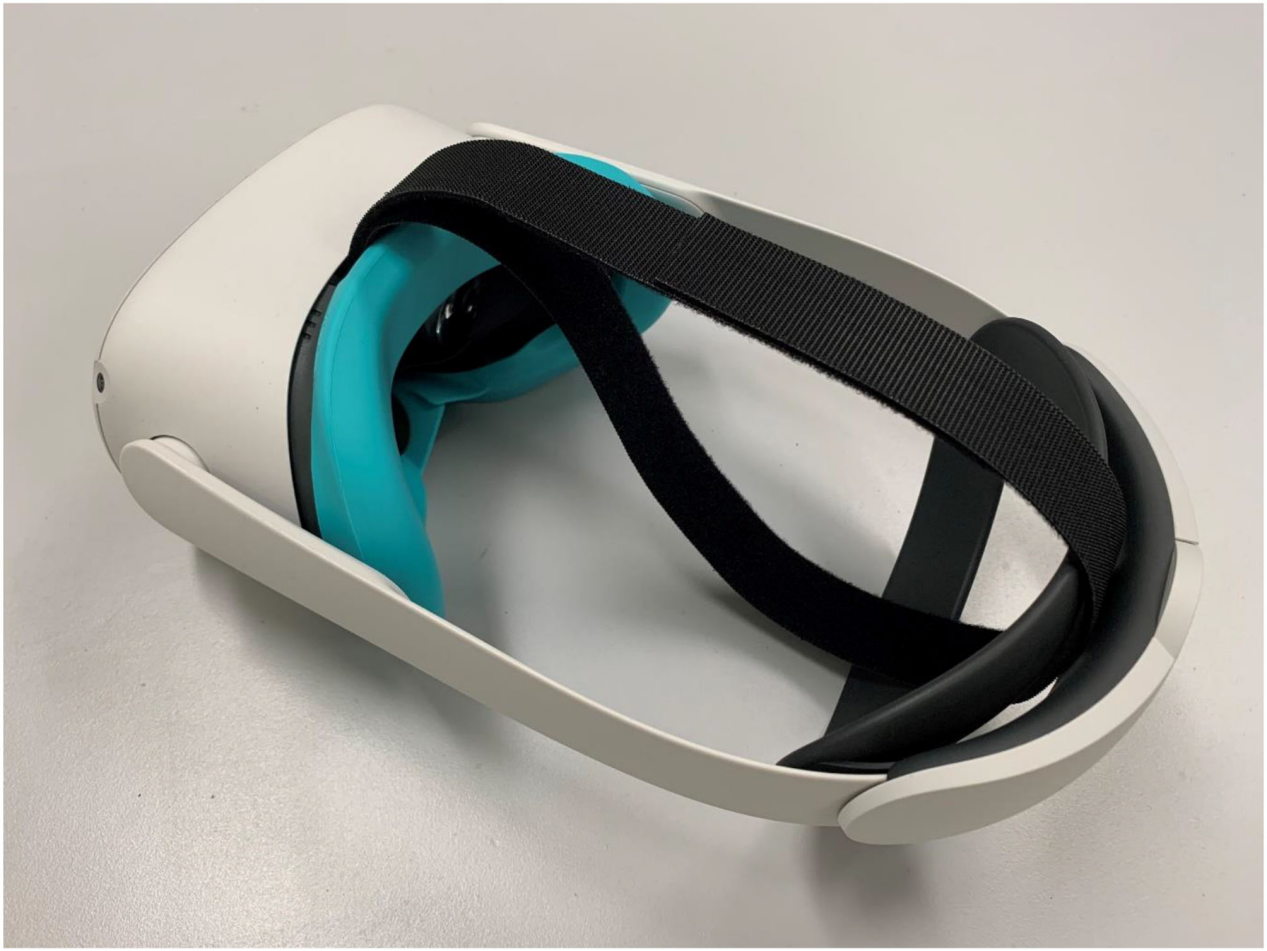
Oculus Quest 2 with non-absorbent wipeable facial interface with replaceable silicone cover and replaceable and washable Velcro securing strap.

VR HMDs are classified as non-critical items as per Spaulding classifications i.e., objects that contact intact skin but not mucus membranes, require cleaning and low-level disinfection (14). Our guideline recommends that the VR components that are difficult to clean or cannot be cleaned be replaced with cleanable or single use components. One example is the fabrics on the main body of the HMD and Velcro based adjustment on the head straps. Most VR models use Velcro based fastening systems and have porous facial interfaces posing cleaning and disinfection challenges. The inclusion of these materials limits the ability for effective cleaning and disinfection of the headsets between use. We suggest these Velcro based elements should be replaced or covered with a cleanable product. Incorporating advice to clean shared equipment between users from the Clinical Excellence Commission “Cleaning of the Healthcare Environment Policy Directive” (15), we recommend all other cleanable parts should be wiped with neutral detergent and due to the proximity to mucous membranes followed by disinfection with a manufacturer approved product. Additionally, and in order to reduce the potential risks associated with shared HMDs, we recommend replacing the absorbent foam based facial interface pads with non-absorbent wipeable facial interfaces (16) as seen in Figure 1 or disposable facial interfaces. When making decisions to retrofit HMDs the original design of the headset should be used as a guiding principle to maintain user comfort. Consulting designers could help address fit and design issues, doing this work early could help reduce complexity and issues in human-technology interaction when scaling the revised HMDs in this situation.

The use of UVC light has been shown to be an effective method of disinfecting surfaces (17). A number of manufacturers have recently launched commercial devices specifically targeting the disinfection of HMDs using UVC light (18, 19). These UVC disinfection devices are beginning to be adopted into some industry, academic, and healthcare settings. There are challenges associated with using UVC light for the disinfection of HMDs. The UVC light units can be expensive and the process requires adequate time for the UVC light to disinfect effectively. Additionally, the varied design of HMDs means that there can be shadows or covered sections of the equipment which the UVC light does not reach without adequate loading of the HMDs into the unit. These challenges further highlight the need for effective, feasible and scalable cleaning processes with neutral detergents to form the basis of any guidelines (20). In conjunction with adequate cleaning processes UVC light disinfection devices could form a part of a cleaning and disinfection process.

In addition to mitigating measures discussed above, our guideline takes into consideration preventative measures. These include multiple iterations of hand hygiene before, during and after use, not using the device if user has cold or flu like symptoms or any non-intact skin to prevent any breach in infection prevention and control principles.

### Summary Step by Step Guideline for VR Cleaning and Disinfection

We recommend the following steps for preventative measures and VR HMD cleaning and disinfection in between use. All cleaning and disinfection should be completed using a Therapeutic Goods Administration (TGA) and manufacturer approved disinfectant.

- Perform hand hygiene.
- Remove disposable face pads (interface) and discard.
- Clean reusable face pads with a detergent solution or wipe.
- Clean hands with alcohol-based hand rub or soap and water.
- Use a new wipe to clean the inner surface of HMD.
- Using a new wipe, clean the outer surface.
- Clean the handheld devices with a new detergent wipe.
- Disinfect the reusable components of the headset.
- Leave the items to dry and store them in a clean sealable and disposable bag.
- Perform hand hygiene after completion.

The full guide can be accessed via the Clinical Excellence Commissions website Cleaning and disinfection of virtual reality equipment (13).

## Discussion

The need for the resumption of educational activities in a variety of settings during a pandemic has provided a catalyst for accelerated innovation and development. The NSW experience of ensuring that education through VR HMD could be safely resumed has had a number of positive and unforeseen impacts. Previously unrecognized infection risks were identified as a result of heightened awareness driven by the pandemic. Although, disposable aspects of HMDs would be an optimal approach to minimize infection risk and logistical concerns, the current generations of HMDs do not allow for this approach and potentially disposable options such as Google cardboard devices do not provide the same levels of interaction or immersion. We suggest that HMD manufacturers be guided by Infection Prevention and Control principles in future design iterations.

We developed a rapid solution in the form of a guideline through the collaboration of experts working across multiple sites, governmental organizations and industry. This “COVID-19 silver-lining” has enabled safer research and education practices through the combined efforts of numerous parties who traditionally might not have worked together to problem solve. We suggest that this learning is relevant to many areas in healthcare and that we should continue finding opportunities for innovation during crisis in other areas of practice. Future direction for this work involves testing the efficacy of the proposed guideline which is currently rolled out across NSW, Australia.

## Author Contributions

NM: lead author. KD and SJ: IPAC content expertise, guideline development, and review/support. NM and PH: educational and leadership content expertise and review/support. NM and PP: VR and microbiology content expertise and review/support. RS: IPAC, infectious diseases content expertise, and review/support. NA: VR based education expertise and publication review/support. All authors contributed to the article and approved the submitted version.

## Conflict of Interest

The authors declare that the research was conducted in the absence of any commercial or financial relationships that could be construed as a potential conflict of interest.
